# Viral Induced Effects on a Vulnerable Epithelium; Lessons Learned From Paediatric Asthma and Eosinophilic Oesophagitis 

**DOI:** 10.3389/fimmu.2021.773600

**Published:** 2021-11-29

**Authors:** Rebecca L. Watkinson, Kevin Looi, Ingrid A. Laing, Antonella Cianferoni, Anthony Kicic

**Affiliations:** ^1^ Division of Paediatrics, Medical School, The University of Western Australia, Nedlands, WA, Australia; ^2^ Wal-Yan Respiratory Research Centre, Telethon Kids Institute, Perth, WA, Australia; ^3^ School of Public Health, Curtin University, Bentley, WA, Australia; ^4^ Pediatrics Department, Perlman School of Medicine, University of Pennsylvania, Philadelphia, PA, United States; ^5^ Centre for Cell Therapy and Regenerative Medicine, School of Medicine, The University of Western Australia, Nedlands, WA, Australia

**Keywords:** rhinovirus (RV), epithelium, asthma, wheezing, allergic, airway, paediatric, EoE

## Abstract

The epithelium is integral to the protection of many different biological systems and for the maintenance of biochemical homeostasis. Emerging evidence suggests that particular children have epithelial vulnerabilities leading to dysregulated barrier function and integrity, that resultantly contributes to disease pathogenesis. These epithelial vulnerabilities likely develop *in utero* or in early life due to various genetic, epigenetic and environmental factors. Although various epithelia are uniquely structured with specific function, prevalent allergic-type epithelial diseases in children potentially have common or parallel disease processes. These include inflammation and immune response dysregulation stemming from atypical epithelial barrier function and integrity. Two diseases where aetiology and pathogenesis are potentially linked to epithelial vulnerabilities include Paediatric Asthma and Eosinophilic Oesophagitis (EoE). For example, rhinovirus C (RV-C) is a known risk factor for paediatric asthma development and is known to disrupt respiratory epithelial barrier function causing acute inflammation. In addition, EoE, a prevalent atopic condition of the oesophageal epithelium, is characterised by similar innate immune and epithelial responses to viral injury. This review examines the current literature and identifies the gaps in the field defining viral-induced effects on a vulnerable respiratory epithelium and resulting chronic inflammation, drawing from knowledge generated in acute wheezing illness, paediatric asthma and EoE. Besides highlighting the importance of epithelial structure and barrier function in allergic disease pathogenesis regardless of specific epithelial sub-types, this review focuses on the importance of examining other parallel allergic-type disease processes that may uncover commonalities driving disease pathogenesis. This in turn may be beneficial in the development of common therapeutics for current clinical management and disease prevention in the future.

## What Is Paediatric Asthma and Why Is Wheezing Important?

Asthma is a heterogeneous, multifaceted respiratory disorder often emerging in early childhood (1–4). It is considered a symptomatic respiratory disorder, ranging from mild to severe. A review of several birth cohorts and risk factors for asthma development that encompassed 122 paediatric studies identified over 60 individual asthma definitions with different parameters for diagnosis (5). Thus, a clear definition has not been established (6). Clinically, paediatric asthma is diagnosed as having onset between the ages of 0 months up to 18 years, although approximately 80% of paediatric asthma cases begin between the ages of 0 months and 6 years (7). To further improve asthma diagnosis in the paediatric population, the Asthma Predictive Index (API) was developed to assist diagnosis of asthma under 3 years. However, observations from several cohort studies have indicated that asthma diagnosis in children under five years is difficult, with most standard testing regimens being variable or inaccurate under the age of seven (5, 8, 9). Many children, in particular, infants, have episodes of wheezing, which is often associated with respiratory viral illness (10) and has been shown to be a strong predictor for asthma diagnosis (1, 11). However, there is often heterogeneity in asthma onset particularly between gender and age. Males are more likely to be diagnosed pre-puberty and to have a heritable component to their disease (1, 12, 13). Females, in contrast, are often diagnosed with asthma later in life. Irrespective of such gender differences, children with wheezing illness exhibit clinical pathological features including smooth muscle hyper-constriction, immune responses such as inflammation, chronic remodelling such as mucous metaplasia and the resultant symptoms. A large proportion of children with early onset asthma have these innate immune system pathologies of allergic disease, and are also found to have elevated adaptive immune cells such as eosinophils in both blood and inflamed tissue (14, 15). Collectively, these pathological changes are triggered by insults such as acute viral infection or allergens which then contribute to a progressive loss of lung function through repeated damage to the airways. This is particularly evident in children who experience more intense and frequent asthma exacerbations (1, 3, 4).

Children exhibiting severe symptoms or respiratory distress caused by several of these pathologies may be hospitalised as their illness requires intervention (16–20) and in rare cases may be life-threatening (21–23). The frequency and severity of wheezing illnesses in infancy and early childhood may determine the likelihood of paediatric asthma development. Paediatric asthma is characterised by multiple phenotypes that have been identified in several different paediatric birth cohort studies (2–4, 24, 25). The Tucson Arizona birth cohort (1) has examined factors affecting presentation of wheezing illness before three years of age in relation to wheezing illnesses at six years and identified four specific phenotypes, (A) non-wheezing illness, (B) intermittent wheezing illness, (C) late-onset wheezing illness and (D) persistent wheezing illness (**Table 1**). This seminal study (1) suggests inherent differences in likelihood of asthma development disease phenotype and the likely mechanisms of disease progression.

**Table 1:** Asthma Phenotypes identified in the tucson arizona birth cohort and corresponding lung function findings (1).

Asthma Phenotypes Identified in Tucson Arizona Birth Cohort (1)	Children in study assigned to phenotype (%)	Relevant Findings
Children who had never wheezed by six years	51.5%	N/A
Children who had at least one LRTI/wheezing in the first three years of life but none at six years	19.9%	Decreased airway function by the age of one year and at six years
Children who had non-wheezing before three years, but had wheezing at six years	15%	N/A
Children who were wheezing both before three years and at six years	13.7%	Normal lung function under the age of one year, decreased lung function at six years

Table showing distinct asthma/wheezing phenotypes in children in the first six years of life adapted from the Asthma and Wheezing in the First Six Years of Life paper utilising the Birth Cohort from Tucson Arizona by Martinez and Colleagues.

To address this further, Oksel et al. (25) used latent class analysis to assess five other birth cohorts and found that the ‘persistent wheeze’ phenotype (26) has the strongest association with asthma development (25). In addition, all asthma phenotypes in which wheezing illness was present had significantly diminished lung function by 4 to 5 years of age when compared to non-wheezing children (25). However, observations between groups suggest that some children are uniquely susceptible to asthma development, and that in addition to symptoms such as wheezing, other factors from the prenatal to early childhood period contribute to the persistent wheezing phenotype and asthma susceptibility. For example, using multivariate analysis, Hallit and colleagues (4) found that early persistent wheezing at one year of age was independently associated with respiratory distress, excess bronchial secretions, reflux and nocturnal cough at two months of age (4). Others have found that maternal smoking during pregnancy and maternal history of asthma are also associated with the early persistent wheezing phenotype in children from two months to one year of age (4, 13, 27). Furthermore, the incidence rate of wheezing is increased when these same risk factors are paired with paternal history of asthma and cutaneous rash at two months of age (4, 27). In addition, another contributing factor for asthma development is allergic sensitisation to at least one allergen. Rubner and colleagues (28) have reported that 65% of children that were sensitised under twelve months, ended up being diagnosed with asthma by 13 years (28), and others have found that allergic sensitisation by the age of three is pivotal in asthma development (1, 3). Through the Urban Environment Childhood Asthma (URECA) birth cohort (24), a study characterising patterns of wheezing and allergic sensitisation in early life, five wheeze and atopy phenotypes were identified: (1) low wheeze/low atopy; (2) low wheeze/high atopy; (3) transient wheeze/low atopy; (4) high wheeze/low atopy; and (5) high wheeze/high atopy (24). Although asthma was overrepresented in the high wheeze phenotypes, most cases of respiratory morbidity were observed in children with both frequent (persistent) wheezing and allergic sensitisation (high atopy) (24). Children that are pre-disposed to airway vulnerabilities through a variety of factors may have more severe and frequent wheezing illnesses in response to an environmental trigger in early life that often leads to an asthma diagnosis. Therefore, recurrent wheezing illnesses in early childhood are a potential indicator of eventual paediatric asthma diagnosis. Furthermore, the combination of exogenous insults to the airway in early life, and the child’s specific innate and adaptive immune responses to these may further potentiate any vulnerabilities, and likely contribute to asthma development.

Collectively, numerous heterogeneous risk factors contribute towards asthma susceptibility as well as wheezing illness in children. With regards to asthma development, parental history of asthma, host genetics, wheezing illnesses and wheeze phenotype all play a pivotal role as well as exposure to allergens, pathogens and exogenous particulates (1, 4, 19, 20, 29–31). Additional risk factors that contribute to wheezing illnesses and asthma exacerbations in susceptible children include exhaust fumes, cold air, and respiratory viruses known to cause acute respiratory infections (ARI). Respiratory viral infections such as respiratory syncytial virus (RSV), influenza, adenovirus, coronavirus and rhinovirus (RV) have also been highlighted as key triggers of wheezing illnesses and asthma exacerbations in children (18–20, 32–37). Furthermore, various studies have suggested a potential nexus between the airway epithelium, respiratory viral insults and its association with wheezing illness. However, the mechanism for this remains unclear (20, 37–39) but there is cogent evidence (40–45) to support the pivotal and contributory role of the airway epithelium to disease progression.

## An Excellent Defence – How Does the Epithelium Enact Its Integral Function?

**Figure 1:**
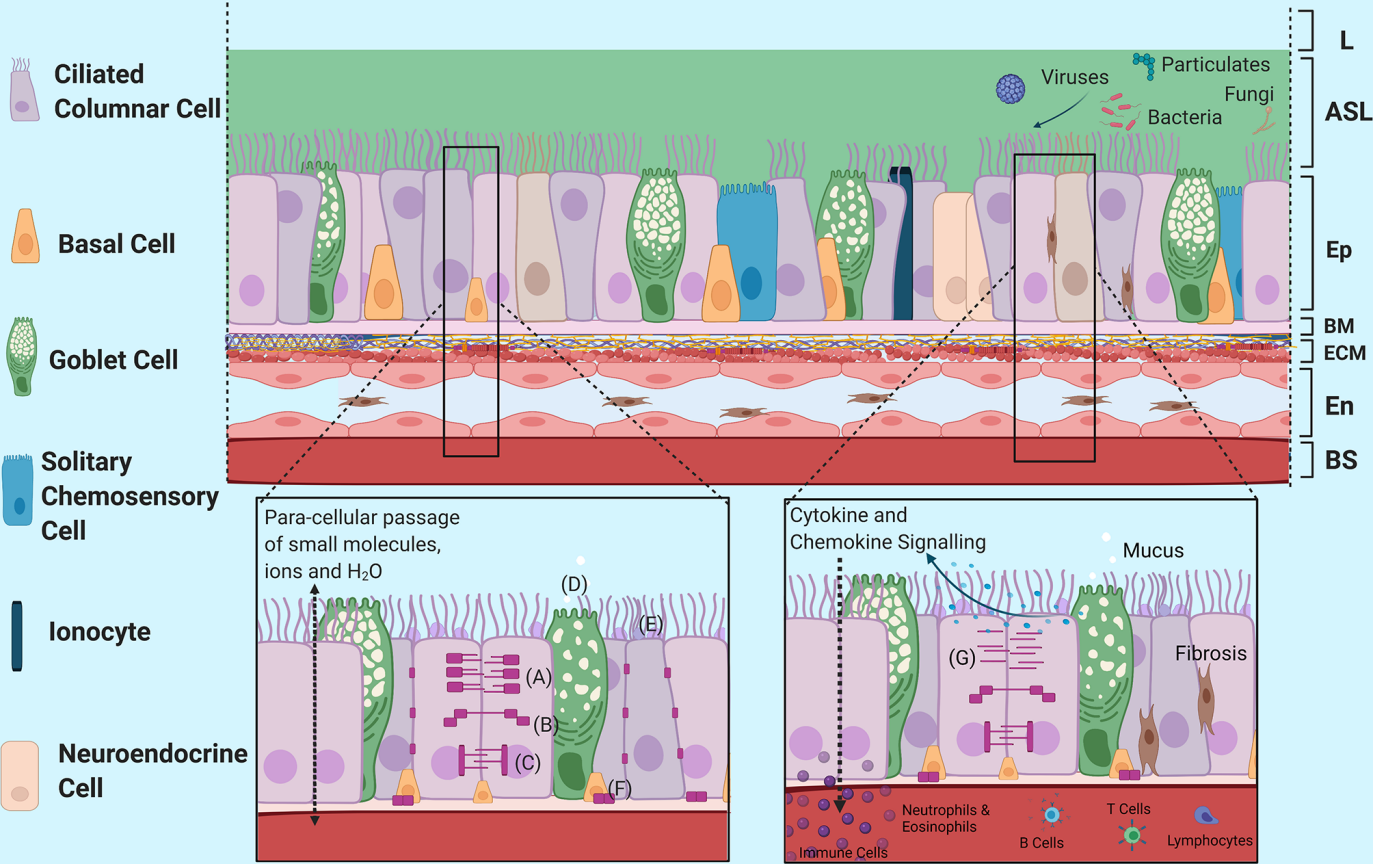
Structural Morphology and Barrier Functions of the Airway Epithelium. An overview of the structural morphology of the airway epithelium and it’s multifaceted barrier functions including structural, mucociliary and immunomodulatory barrier functions in response to injury. (A) Tight Junction Complex, (B) Adherence Junction Complex, (C) Desmosomes, (D) Mucin Release into Airway Surface Liquid, (E) Structural Mucins, (F) Hemidesmosomes, (G) Disruption of Junctional Complexes due to epithelial vulnerabilities and injury. L, Lumen; ASL, Airway Surface Liquid; Ep, Epithelium; BM, Basement Membrane; ECM, Extracellular Matrix; En, Endothelium; BS, Bloodstream. Created with BioRender.com.

The airway epithelium is a pseudostratified structure whose complex functions provides protection through structural, mucociliary and innate-immunological barriers. These barriers work synergistically in maintaining epithelial homeostasis and providing a dynamic response to pathogens, allergens and particulate matter. As reviewed by Knight and Holgate (46) and also identified by Garcia and colleagues (47), there are various types of epithelial cells, including basal, club, ciliated columnar and goblet cells (46–48). In addition, there are other lesser known epithelial cells involved in innate-immunological epithelial function including neuroendocrine cells, ionocytes expressing CFTR that contribute significantly to the muco-viscosity of airway surface liquid, and solitary chemosensory cells which are involved in the detection and release of neurotransmitters and ion channel function, among other functions (49–51). The heterogeneity of epithelial cell populations is further highlighted when comparing between the proximal and distal airway epithelium, each having similar yet distinct roles and functional processes (**Figure 1**).

The airway epithelium was once regarded as inert but is now known to be a dynamic barrier which actively regulates the passage of smaller molecules, ions and water while remaining impermeable to macromolecules (**Figure 1**). This is achieved *via* a myriad of junctional complexes (52, 53), which provides anchorage not only between adjacent cells but also to the basement layer and works to prevent exogenous molecules from passing through to the systemic circulation (52–55). In addition, O-glycosylated linear glycoproteins, which are mucins produced and secreted by goblet cells onto the airways, provide further defence against external insults. Structural mucins exist as brush-like structures attached to epithelial cells adjacent to the lumen in between cilia to form an additional structural barrier. These mucins bind to or block pathogens and other exogenous stimuli to prevent them from reaching the cell surface (56–58). Goblet cells, along with non-epithelial submucosal glands, also secrete non-structural polymeric mucins such as MUC5AC and MUC5B that form interconnected strands and sheets as part of airway mucus (59, 60). Airway mucus forms a percentage of the airway surface liquid layer atop the epithelium and helps to capture exogenous matter including pathogens and particulates (59, 60). Mucociliary clearance of the trapped pathogens and particulates are then unidirectionally propelled away from the distal and proximal airways towards the oropharyngeal regions for expectoration.

Adding to the protective capacity provided by mucins, basal epithelial cells have also been demonstrated to have a reparative capacity (61–63). Following wounding or injury to the epithelium by airborne pathogens, initiation of the cell repair process commences with the leading edge basal cells surrounding the injury site migrating into the wound to begin restitution of barrier integrity. This then follows by proliferation and differentiation as part of the wound repair process (61).

Complementing the physical defence of the airway epithelium, airway epithelial cells have also been shown to have an innate-immunological function that often leads to the recruitment of the adaptive immune system. This encompasses epithelial derived cytokines (**Table 2**) that define the pro-inflammatory responses that comprise part of the innate immune system and are released in response to stimuli. These have been comprehensively reviewed in numerous studies assessing adult airway epithelium (106–108) and will not be covered within the scope of this review.

**Table 2:** Various cytokines involved in immune response to different stimuli.

	Particulate Matter	Viral Infection	Bacterial Infection	Fungal Infection
**Cytokines associated with immune response to:**	TNF-α (64, 65) IL-1β (64–67) IL-6 (64–66, 68) IL-8 (66–68) IL-10 (69) MIP-1 α (64) GM-CSF (64, 66, 67) LIF (66, 67) TLRs (70)	IFN-α (71, 72) IFN-β (71, 73, 74) IFN-γ (71, 75, 76) IFN-λ (77, 78) IL-1β (79, 80) IL-6 (79, 81, 82) IL-8 (79, 83–85) RANTES (84, 85) GM-CSF (84) TNF- α (70, 82) CXCL8 (81) CXCL10 (81, 86) TSLP (87) MCP-1 (82)	IFN-γ (88–90) IFN- γ-IP10 (91) IL-1 α (92) IL-1β (91–93) IL-6 (91, 92, 94) IL-8 (92, 94) GM-CSF (95) G-CSF (95) TNF-α (92, 94) NFκB (96) MCP-1 (91, 92) MIP-1 α (91) RANTES (91) TLRs (97–99)	TLRs (100, 101) IL-1β (102) IL-2 (103) IL-4 (103) IL-6 (102) IL-10 (103) IL-12 (103, 104) IL-18 (104) IL-36 (101) TNF-α (102–105) IFN-γ (103, 104)

Various cytokines that are produced or recruited upon insult or injury by different stimuli on the airway epithelium, including particulate matter, viruses, bacteria and fungi.

The paediatric upper airway epithelium has been shown to have 91% transcriptional homology with the lower airway epithelium and a similar gene expression profile particularly in children with atopy (109). This conservation between the upper and lower respiratory airways lends support to the unified airway hypothesis that disease manifestation at both sites is likely a consequence of similar processes (109). However, it is acknowledged that this may not always translate functionally in both upper and lower respiratory airways. Foxman and colleagues (110) have shown that there is a temperature dependent innate immune response in murine airway epithelial cells infected with RV-A1. At 37°C, which is equivalent of the lower airways, there were increased interferon (IFN) type I and III genes, along with IFN stimulated genes compared to at 33°C, the equivalent of upper airways. This suggests a higher level of anti-viral defence or a more potent host response to infection at the higher temperature and in the lower airway. Furthermore, Lopez-Souza and colleagues (111) identified that RV-A16-infected human bronchial epithelial cells at air-liquid interface had lower trans-epithelial resistance, increased viral load (20-30 times), increased anti-viral and inflammatory cytokine production such as regulated on activation, normal T-cell expressed and secreted (RANTES), interleukin (IL) -8, IP-10, and IL-1α, compared to RV-A16-infected human nasal epithelial cells irrespective of asthma diagnosis (111). This suggests that the nasal epithelium has a greater protective capacity than the bronchial epithelium, and that asthmatic children with epithelial vulnerability may have a greater chance of RV infection spreading from the upper to the lower respiratory tract. Foxman et al. also found that genetic deficiency in mitochondrial antiviral-signalling protein (MAVS) and IFN type 1 receptor allowed for higher levels of RV replication at 37°C (110), suggesting that children with innate immunity deficiencies such as inadequate IFN response in the airway may allow for greater severity of RV infection. However, not all children with asthma are deficient in IFN production, as shown by Miller et al., where some children were able to produce sufficient IFN even while exacerbating (112). It was also demonstrated that IFN-λ was increased in nasal lavage fluid from wheezing asthmatic children with an RV infection compared to non-wheezing asthmatic children with an RV infection (112). Therefore, this again suggests that there are immunological and mechanistic differences between asthma phenotypes in different children, as well as potential respiratory epithelial differences, resulting in varying levels of vulnerability towards exogenous stimuli.

## What Is a Vulnerable Epithelium and How May This Vulnerability Occur?

It is evident that despite similarities between the upper and lower airways of a child, there may be innate differences compared to other children. Factors that can cause this heterogeneity include host genetics (29, 113–117), epigenetic modifications throughout infancy and early childhood (117–119) as well as environmental risk factors (61, 120–122), that together, result in variable gene expression patterns and clinical characteristics. These may be hereditable or caused by environmental modification during the *in utero* or early childhood period. Although children with a healthy, respiratory epithelium functioning at full capacity are unlikely to develop vulnerability, those who are at risk or predisposed to disease may be more susceptible to external insults such as viral infection, allergens, or particulate matter by exhibiting an atypical epithelial barrier and immune response. These insults may then contribute to further epithelial dysregulation.

Studies have observed differences in individual genes involved in innate immune responses activated during viral respiratory illness including *JAK2, STAT4, MX1, DDX58, VDR* and *EIF2AK2* (29, 114). Interestingly, genetic polymorphisms on these genes have been significantly associated with asthma exacerbations (29, 114). Variations in the *ORMDL3/GSDMB locus, and GSDMB, CD14, CC16, CYSLTR1, ST2, GSTP1, and IL1RL1* genes among others (123–129), some of which may be epithelium and innate immunity specific have also been shown to be associated with childhood onset asthma. In addition, single nucleotide polymorphisms (SNP) have been shown to occur in mucin genes, potentially causing alterations in airway mucus (130). SNPs such as the *MUC5AC coding rs1132440 G/C* (C risk allele variant) have been associated with increased *MUC5AC* expression during respiratory viral illnesses (130). Jackson and colleagues found that this particular risk allele enhanced expression of a gene hub containing *MUC5AC* and other genes related to mucus hypersecretion and activation of eosinophils resulting in clinical outcomes such as airway-hyper-responsiveness, mucus plugging and increased inflammatory processes (130).

Other genetic variations in cellular viral receptors are also likely to contribute to epithelial vulnerability. SNPs such as *rs5498* and *rs688* have been found in RV species A and B receptors - the intracellular adhesion molecule (ICAM-1) and the low density lipoprotein receptor (LDLR) respectively (131, 132). However, the most clinically relevant viral receptor SNP *rs6967330 A/G* (*Cys529Tyr*) is located in the cadherin related family member 3 (*CDHR3*), the viral receptor for RV-C, the species most prevalent in paediatric wheezing illness (19, 20, 35–37, 115, 116). The CDHR3 receptor protein is present on all ciliated epithelial cells with its extracellular domains 1-3 mediating the interaction of epithelial cells with RV-C (133). The *CDHR3* gene risk allele is associated with a ten-fold increase in cellular CDHR3 expression (134) and has been shown to increase RV-C infection levels and protein surface localisation of the receptor on epithelial cells potentially leading to more severe infection (134). This risk allele has also been associated with earlier, faster ciliogenesis and a ten-fold increase in *FOXJ* transcription factor expression, a known driver of basal epithelial cell differentiation into ciliated cells (116, 117). Despite this, it appears to have no effect on ciliary beat frequency or the integrity of tight junctions (117). Furthermore, cleaved cytoplasmic domains of some cadherins are able to self-activate their own gene expression which decreases as cells mature and protein interactions increase (135). This suggests that epithelial cell damaged due to infection may trigger the repair process, which in turn may lead to an increase of immature cells and increased CDHR3. RV-C infection has been observed to decrease *CDHR3* mRNA expression in wheezing children compared to the control group (136). Clinically, the *CDHR3* risk allele has been associated with increased risk of wheezing illness leading to hospitalisation. It was found to be overrepresented in wheezing children, and children with this risk allele were observed to need an increased amount of respiratory medical care during wheezing illness (136).

There are various genetic risk factors that may cause variability in the amount gene encoding or gene function as recently identified by Khoo and colleagues in their Mechanisms of Acute Viral Respiratory Infection in Children (MAVRIC) acute wheezing cohort (113). They found that some children, in response to an acute viral-induced wheezing illness, have different upper airway gene network expression patterns in their anti-viral Interferon Regulatory factor 7 (IRF7) network. These network patterns, named “IRF7hi” and “IRF7lo”, are likely to represent different immune responses to respiratory viral infection. Children characterised as “IRF7hi” exhibited a gene expression pattern associated Th1 and type 1 interferon responses to viral-induced asthma exacerbations. Alternatively, children characterised as “IRF7lo” exhibited a gene expression pattern associated with epithelial cytokine signalling, upregulated growth factor signalling and downregulated anti-viral interferon gamma. The study also reported that children with “IRF7lo” expression exhibited symptoms twice as long prior to hospital presentation from initial symptoms and had cough three times as long compared to children with “IRF7hi” expression. In conjunction, the odds ratio for hospital admission for children with “IRF7lo” was increased by a factor of four and a much shorter time until wheezing illness recurrence. Thus, children with the “IRF7lo” phenotype may have more frequent wheezing illnesses which may potentiate asthma development. These molecular sub-phenotypes may impact on each child’s epithelial response due to the genes and cytokines associated with them.

In addition to genetic risk factors, each child will likely incur epigenetic modifications, due to environmental factors such as diet and exposure to allergens and exogenous pathogens to their airway epithelium during development. These epigenetic modifications have the potential to change epithelial functionality and integrity, as shown by Lund et al., where RV infection causes DNA methylation (118) and has been found to occur in genes including *SMAD3*, that encodes for the cell signalling protein SMAD3, as well as *DDO* and *METTL24*, genes that encode for peroxisomal flavoprotein and methyltransferase 24 respectively (118, 137). Furthermore, Pech and colleagues found that DNA methylation and resultant changes in mRNA expression in genes occurred in nasal cells from children with asthma following infection with RV (119) And that these altered genes are associated with host immune response to viral infection as well as asthma pathogenesis (119).

In addition to genetic and epigenetic risk factors, there are also environmental risk factors. An important subset of environmental risk factors for epithelial vulnerability are those imparted by pre-term birth (<32 weeks) and its associated medical stresses such as oxygenation, steroids use and mechanical ventilation (120). Hillas and colleagues observed that cultured primary nasal epithelial cells from healthy full-term children were able to complete *in vitro* wound closure by 60 hours (120). In contrast, pre-term infants appear to have either (1) delayed but complete (>80%); (2) significant but incomplete (50-80%); or (3) fully incomplete (20-50%) epithelial wound closure (120). Moreover, epithelial vulnerability in pre-term children may develop *in utero* during maternal exposure to pathogens, particulates and resultant inflammation. Chorioamnionitis, an inflammatory *in utero* risk factor for pre-term birth (121, 122), has been associated with increased airway epithelial apoptosis (121) with further studies also showing that epithelial cells treated with bronchoalveolar lavage fluid from infants born pre-term with chorioamnionitis have a reduced capacity for epithelial wound repair after mechanical wounding (122).

A dysregulation in epithelial wound repair potentially leaves children more susceptible to exogenous insults and further epithelial vulnerability. Defective epithelial wound repair has been found to be highly prevalent in children with asthma (38, 39). Deficient production of fibronectin, an epithelial extracellular matrix protein, may contribute to aberrant wound repair (138) although treating *in vitro* cell cultures with exogenous fibronectin only partially restored wound repair (138). Iosifidis et al., using paediatric asthmatic primary airway epithelial cells, demonstrated an aberrant wound migration pattern associated with decreased integrin α5β1 expression (61) that is regulated by the PI3K/Akt pathway (61). The transcriptomic signature associated with aberrant wound repair and the PI3K/Akt pathway was associated with viral-induced wheezing illness, suggesting that RV infection could disrupt the PI3K/Akt pathway particularly in children susceptible to asthma. Importantly, they showed that Akt restoration with a repurposed drug resulted in improve epithelial repair capacity and integrin expression, thus providing proof of principle that a dysregulated airway epithelium can be therapeutically targeted (61).

There are many other risk factors that increases epithelial vulnerability and eventually leading to the development of persistent wheeze and ultimately, asthma. Epithelial vulnerability has the potential to cause a dysregulated response to an environmental insult which may potentiate disease progression. Furthermore, it is possible that RV infections, particularly RV-C, may infect the vulnerable epithelium opportunistically. The consequence of this may be a repetitive cycle of infection, dysregulated epithelial barrier function, repair, leading to persistent wheezing exacerbations and potential asthma pathogenesis. Although there has been progress in research on epithelial vulnerability and its potential to contribute to airway disease pathogenesis, elucidating the role of the vulnerable epithelium in disease progression and in particular, its association with respiratory viral infections such as rhinovirus, remains significantly inadequate.

## The Significance of Rhinovirus – What Have Clinical and Laboratory Based Studies Identified So Far?

Rhinovirus (RV) is the most common virus found in children, particularly in those admitted to hospital with wheezing (19, 139). As reviewed by Palmenberg and Gern (140), RVs are viruses that are part of the enterovirus genus and the picornaviridae family. These viruses are known to have a five prime (5’) virus encoded protein (VP) and a three prime (3’) poly adenosine tail like that of messenger RNA (140). They are small, approximately 30nm in diameter irrespective of RV species. Three known species of RV exist: RV-A, RV-B and RV-C. There are three host viral receptors for RVs; major genotypes of RV-A and RV-B use the intracellular adhesion molecule 1 (ICAM-1), and minor genotypes use the low density lipoprotein receptor (LDLR) (140), however, RV-C uses CDHR3 as its receptor (134, 141, 142). There are many RV strains with more than 150 RV sub-types identified and extensive antigenic diversity and presently, there are no therapeutics capable of mitigating its effects and the development of serious respiratory disease (143). Interestingly, the increase in RV infections in spring and autumn correlate with hospital admission rates for paediatric asthma exacerbations (144, 145) and the virus is present in the majority of paediatric asthma exacerbations (11, 19, 20, 23, 35–37, 144, 146–150). The anti-viral immune response triggered by ARIs is likely to contribute to the overall inflammatory load that is in turn capable of triggering an asthma exacerbation.

The inter-relationships between RV infections, epithelial vulnerability and why some children have persistent wheeze, leading to the development of asthma, is still largely unclear (146, 151). Rhinovirus is commonly detected in children with ARIs (11, 139, 152–160), with ARIs leading to symptoms in the lower respiratory tract being a major cause of paediatric morbidity and mortality worldwide (151). ARIs, particularly those caused by RV may contribute towards epithelial vulnerability, further wheezing illnesses and asthma development. RVs are ubiquitous in the community, and it is estimated that they cause ~50% of all upper respiratory tract infections in humans, as well as being associated with acute asthma exacerbations in both children and adults (11, 19, 20, 23, 35–37, 144, 147–150). This is due to RV being identified as the most common virus in wheezing children admitted to the hospital emergency department, associated with 20 - 87.5% of hospitalisations (19, 20, 35–37, 144, 146, 150) and 10 - 15% of admissions into the paediatric intensive care unit (18, 19). The highest instances of RV infection have been reported in children with a history of asthma related symptoms (144). Moreover, Jackson and colleagues utilising the COAST high-risk birth cohort of children with parental history of asthma or allergy, also found that 90% of wheezing illnesses were of viral origin, and that by the age of three years, RV-associated wheezing had a three-fold stronger association with increased asthma risk by six years than aeroallergen sensitisation or RSV (11). Almost 90% of the children that had RV induced wheezing illness at the age of three years were diagnosed with asthma at the age of six (11). They concluded that among all outpatient viral wheezing illnesses in infancy and early childhood, those caused by RV were the most significant predictors of asthma development in their high-risk birth cohort (11). Wheezing illnesses and their relation to house dust mite allergy and RV was also examined in another study, which found that the probability of acute wheezing in children was positively correlated with increasing IgE titres to house dust mite. This was also significantly further potentiated when an RV infection was present during exacerbation (161).

The effect of RV on the paediatric airway epithelium - and its contribution to wheezing illness and eventual asthma development - is not yet fully understood. One study suggests that receptor specificity determined by the major or minor genotype of RV- A and RV-B can influence host response to the virus (162). Other studies have shown that RVs can dysregulate epithelial barrier function and integrity, potentially contributing to asthma development by altering epithelial barrier function and integrity in several ways including by disruption of homeostatic and dynamic cytokine production, tight junction complexes, as well as dysregulating wound repair (38, 79, 144, 163).

An example of one of the effects that RV has on the epithelium is that RV is able to dysregulate the production of anti-viral and pro-inflammatory cytokines and biochemical signalling molecules during infection, particularly in asthmatic children (144, 164–169) potentially leading to systemic inflammation and the switch from the innate immune system of the epithelial response to the adaptive immune system. Contradictory findings have been made around the interferon response in this setting. For example, some have found that IFN type I, II and III genes are induced by the airway epithelium during RV infection (71). Interestingly, this response appears to be dysregulated particularly in asthmatics (170). IFN-α levels have been found to be upregulated in PBMCs from asthmatic children infected with RV, while IFN-λ is already upregulated at baseline in these children (171), which, when combined, suggest a hyperactive immune response. In addition, Miller and colleagues (112) found that IFN-λ is further increased in RV-infected-wheezing children with asthma compared to RV-infected-non-wheezing children with asthma (112). Conversely, others have shown that primary airway epithelial cells from asthmatic children infected with RV-A1 and RV-A16 produce less IFN-β and IFN-λ than cells from their healthy non-asthmatic counterparts (38, 162, 170, 172). In addition, Edwards and colleagues (170), found that deficient IFN β and λ was positively correlated with asthma severity, with high levels of IFN deficiency found in severe therapy resistant atopic asthmatics (170). However, the controversy surrounding these findings is potentially due to children having different interferon molecular sub-phenotypes (“IRF7hi” and “IRF7lo”) such as was identified in the MAVRIC acute wheezing cohort by Khoo and colleagues (113). These studies collectively suggest a dysregulated anti-viral response to RV infection. In conjunction with epithelial IFN production in response to RV, there are other antiviral mechanisms that appear to be dysregulated during infection, such as the signal transducer and activator of transcription 1 (*STAT*1) signalling pathway. *STAT*1 is typically activated in response to RV in epithelial cells and activates a signalling cascade that promotes the expression of anti-viral genes (71) as part of the multifaceted epithelial response to infection.

The airway epithelium’s multifaceted response to RV infection has been shown to upregulate gene expression and ultimately the release of innate immune system pro-inflammatory cytokines, chemokines (79, 81, 83–86, 173), including eotaxins, IL-17C, IL-4, IL-5, IL-13, IL-33, NFκB, TSLP (71, 85, 173–175) and others (**Table 2**). Nasal washes collected from children during confirmed RV infection, have increased thymic stromal lymphopoietin (TSLP) levels at the time of infection which is also linked to atopy and plays a role in many allergic diseases (176). Kennedy and colleagues (173), observed an increase in TSLP gene expression and protein as well as gene expression of IL-25 and IL-33, in asthmatic donor lungs compared to non-asthmatic controls following RV-A39 infection (173). They also observed that there was only a difference in carbachol-induced airway constriction between the two cohorts post RV-A39 infection, suggesting an altered immune response even in the lower airway (173). In addition, Subauste and colleagues (177) demonstrated that RV-B14 was able to induce TNF-α, IL-6 and IL-8 release in human bronchial epithelial cells, and that prior exposure to TNF-α, increased susceptibility to RV-B14 infections suggesting the potential for cytokines to potentiate further RV infection (177). Another study examining the nasal cytokine profiles of children hospitalised with respiratory wheeze found that RV-C-induced wheezing was identified to have a characteristic Th2 type cytokine release profile in both non-asthmatic children and asthmatic children (17). Interestingly, the same study also found that cytokines IL-17 and IL-1β (characteristic of Th17) were increased in children with pre-existing asthma and not in non-asthmatic children, irrespective of both being diagnosed with wheeze and viral infection symptoms (17). In addition, the pro-Th2 inflammatory profile inducing cytokine IL-33, is shown to be downregulated in children with the “IRF7hi” phenotype defined in the paper by Khoo and colleagues (113) but not in the “IRF7lo” phenotype and thus could potentially be a driver of wheezing illnesses in children with the “IRF7lo” phenotype. IL-33 has been associated with paediatric asthma in other studies also (17, 113, 175, 178). Moreover, in response to RV infection by adult bronchial biopsy specimens, there is an increased number of sub-epithelial inflammatory cells that express IFN, as well as epithelial and sub-epithelial pattern recognition receptors (PRR) (169). It is important to note that RV infection is recognised by PRRs (86, 179, 180) that activate MAPK signalling pathways that in turn induce inflammatory gene production. MAPK pathways p38 and JNK have pivotal roles in the epithelial inflammatory response to RV infection (181). Duel Specificity Phosphatase 10 (DUSP10) also plays a pivotal role by regulating it inflammatory cytokine production (e.g. IL-1β) and has been observed to be downregulated by RV infection, thus weakening the anti-viral response and perpetuating uncontrolled inflammation (181). The overproduction or upregulation of pro-inflammatory cytokines and chemokines potentially confers a hyper-active response which may damage the airway epithelium further and go on to impair barrier function and integrity. Interestingly, even without RV infection present at the time, asthmatic airway epithelial cells from children have been identified to have increased IL-6, epidermal growth factor and prostaglandin-E2 as well as decreased TGF-β1 (39). This suggests intrinsic differences in the airway epithelium between asthmatic and non-asthmatic children, and thus a different response to pathogens such as RV.

RV infection also disrupts the barrier function of the airway epithelium by dissociating tight junction proteins such as zonula-occludens 1 from the tight junction complex in both asthmatic and non-asthmatic children (163, 182). Despite upregulation of the basal gene expression of the tight junctions claudin-1 and occludin in children with asthma compared to non-asthmatics, protein levels are significantly reduced (163). This suggests that although genes are being transcribed, translation into protein may not be occurring. Furthermore, *in vitro* air-liquid interface cultures of epithelial cells established from asthmatic children show a sustained decrease in tight junction protein staining, decreased trans-epithelial resistance, (TEER) and a consequent increase in permeability when infected with RV-A1 (163). Dysregulated tight junction expression and resulting function as seen in asthmatic epithelial cells would potentially allow pathogens and particulates to pass through the epithelial layer and into the bloodstream resulting in a heightened host response.

In addition, RV has also been observed to be able to disrupt epithelial wound repair and increase cellular cytotoxicity (38, 183–185). RV-A1 has been shown to delay wound repair capacity and inhibit apoptotic processes by epithelial cells, exaggerating the already defective repair in the asthmatic airway (38). RV infection of a bronchial epithelial cell line *in vitro* has been able to stimulate mRNA expression and release of basic fibroblast growth factor (bFGF), leading to fibroblastic repair rather than normal epithelial repair processes (184). The release of bFGF is associated with RV-induced cytotoxicity and resultant epithelial necrosis as opposed to apoptosis. Furthermore, RV infection also causes an increase in matrix metalloproteinase (MMP) activity *in vitro*, and may affect proteins of the extracellular matrix to which the epithelium is attached (184). Levels of bFGF and MMP are also induced following RV infection of epithelial cells *in vitro* (184). This is further evidence that epithelial wound repair is dysregulated in asthmatics, and potentiated further by RV. Furthermore, Altman and colleagues (186) examined cellular transcriptome networks and found that peak upregulation of epithelial SMAD3 and type one IFN signalling occurs at day two of a viral-induced asthma exacerbation, followed by peak upregulation of epidermal growth factor, and extracellular matrix at day three to four (186). Deficient or dysregulated wound repair of the epithelium likely leaves the epithelium susceptible to further infection or damage from exogenous insults.

Of the three species of RV, RV-C is of particular interest as it is associated with more severe acute wheezing illnesses (20). In addition, several studies have shown that RV-C accounts for the majority of RV positive cases in children (**Table 3**) (17, 19, 20, 35–37, 188, 189) and coincide with higher asthma severity scores as evidenced in children presenting to the emergency department with a wheezing illness (20). Furthermore, wheezing illnesses induced by RV-C are often more commonly associated with typical asthma symptoms such as wheezing and cyanosis when compared to other RV species (36, 37, 190). It is possible that RV-C may further potentiate epithelial vulnerability, yet this is currently unknown.

**Table 3:** Different species of RV in paediatric patients.

Cohort(s)	Sample Type	Detection Method(s)	RV-A (n)	RV-B (n)	RV-C (n)	Untypable RVs or other viruses (n)	Includes Asthmatics	Ref.
**Adults and Children admitted to hospital (results recorded for children only)** **(Italy)**	Nasopharyngeal Aspirate	Real time RT-PCR	24	6	21	5	Unknown	Piralla et al. (187)
**Children aged 2-16 years with Acute Asthma presenting to hospital emergency department (Australia)**	Nasal Aspirate	Quantitative real-time PCR	31 A or B	31 A or B	76	6	Yes	Bizzintino et al. (20)
**Healthy Pre-school children in the community under the age of 5 years swabbed when presenting with ARI symptoms** **(Australia)**	Nasopharyngeal/Oropharyngeal swabs	Quantitative real-time PCR	99	13	113	13	Unknown	Mackay et al. (188)
**Children <5 years presenting to hospital with an acute wheezing episode (Australia)**	Nasal Samples	Quantitative real-time PCR	38	3	81	13	Yes	Cox et al. (35)
**Children between the ages of 1 month and 14 years admitted to hospital with ARI (Italy)**	Nasopharyngeal Swab	Nuclisens EasyMAG automated extraction system; Quantitative real-time PCR	18	5	22	N/A	Unknown	Esposito et al. (189)
**Children aged 0-18 years presenting to hospital with Acute Wheeze** **(Australia)**	Nasopharyngeal Aspirate, Nasal Swab	Quantitative real-time PCR	85	6	169	N/A	Yes	Hurdum et al. (36)
**Hospitalised children aged 1 month to 16 years and 11 months with lower respiratory tract infection** **(China)**	Nasopharyngeal Aspirate	Quantitative HRV-specific real-time PCR	229	27	100	N/A	Yes	Xiao et al. (190)
**Children hospitalised with Pneumonia (Morocco) 2-59 months**	Nasopharyngeal Aspirate	Quantitative real-time PCR	60	8	89	N/A	Unknown	Annamalay et al. (37)
Children aged 0 to 16 years admitted to a hospital PICU* with ARI** **(Australia)**	Nasopharyngeal Aspirate	Quantitative real-time PCR	40	4	51	N/A	Yes	Cox et al. (19)
**Children <18 years hospitalised with Acute Lower Respiratory Tract Infections** **(Korea)**	Nasopharyngeal Aspirate	Multiplex real-time PCR	55	8	31	N/A	Unknown	Ahn et al. (191)
**Multi-centre *Post-hoc* analysis of Infants under one year of age diagnosed in hospital with Bronchiolitis** **(USA)**	Nasopharyngeal Microbiota	Singleplex real-time PCR	91	12	91	RSV 580	Parental History	Toivonen et al. (146)
**Children (1-59 months) hospitalised with Pneumonia/Controls (Africa)**	Nasopharyngeal/Oropharyngeal swabs	Quantitative real-time PCR assay	199	31	185	N/A	Unknown	Baillie et al. (192)
**Children aged between 24-72 months presenting to the hospital emergency department with respiratory wheeze** **(Australia)**	Nasopharyngeal Swabs	Quantitative real-time PCR	64	N/A	207 Sole Pathogen in 191 of RV-C samples	RSV 42 hPIV 30	Yes	Sikazwe et al. (17)

A table of papers showing the prevalence of RV-A, RV-B and RV-C in different paediatric cohorts. *PICU, Paediatric Intensive Care Unit; **ARI, Acute Respiratory Infection.

Despite anatomical differences, other epithelial surfaces are damaged following insult, which when dysregulated can contribute towards disease manifestation and progression. One example, eosinophilic oesophagitis (EoE), exhibits similar disease characteristics to paediatric asthma including atypical epithelial barrier integrity and dysregulated innate and adaptive immunity (193). Although the intricate interplay between allergic sensitization and airway inflammation has been studied in these diseases (28, 187), their association with epithelial barrier dysregulation remains relatively unknown, however, there is evidence to suggest that systemic inflammation could be a major contributor (191, 192). Therefore, examining parallel epithelial diseases processes may further the understanding of their pathogenesis. For example, to understand the role of the vulnerable epithelium and its dysregulation in acute wheezing illness and paediatric asthma, it is important to examine what is known about the epithelial response to injury in EoE.

## What Can We Learn From Eosinophilic Oesophagitis (EoE) and the Importance of the Epithelium in Driving Atopic Chronic Inflammation?

**Figure 2:**
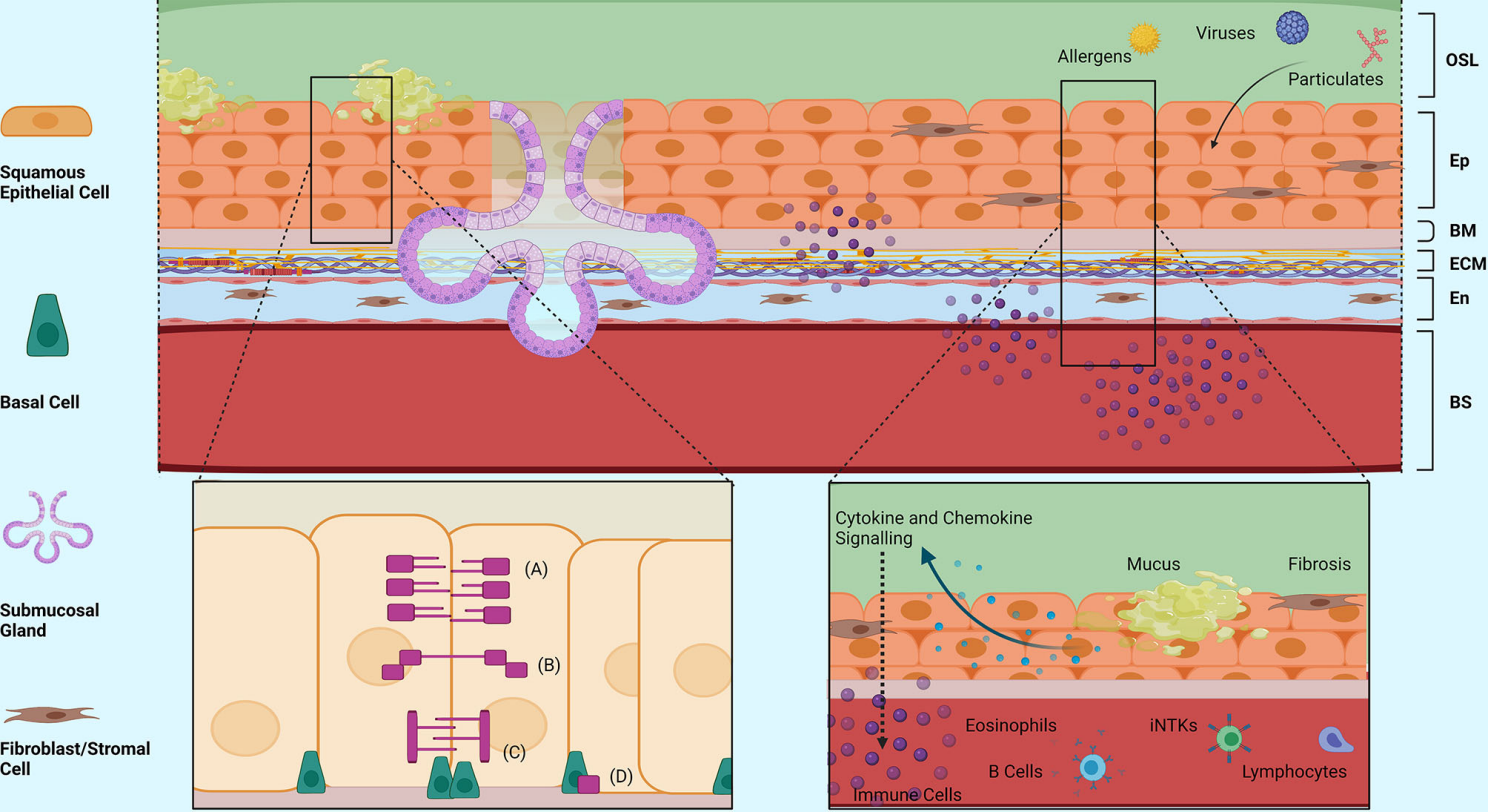
Structural Morphology and Barrier Functions of the Oesophageal Epithelium. An overview of the structural morphology of the oesophageal epithelium and it’s multifaceted barrier functions including structural, mucosal and immunomodulatory barrier functions in response to injury. (A) Tight Junction Complex, (B) Adherence Junction Complex, (C) Desmosomes, (D) Hemidesmosomes. OSL, Oesophageal Surface Liquid; Ep, Epithelium; BM, Basement Membrane; ECM, Extracellular Matrix; En, Endothelium; BS, Bloodstream. Created with BioRender.com.

Eosinophilic Oesophagitis (EoE) is an increasingly prevalent atopic condition defined by eosinophilia of at least 15 eosinophils per high power field (eos/hpf) and symptoms of oesophageal dysfunction (194, 195). As an auto-immune disease, EoE is potentiated by abnormal host response to a trigger, yet still exhibits typical allergy symptoms similar to asthma (**Figure 2**). Although not inherently linked, EoE and asthma are two distinct allergic diseases that have parallel disease processes. Thus, the similarities and differences identified can be drawn upon to expand the knowledge of each disease and further the understanding of the mechanisms behind them. Increasing evidence suggests that genetic predisposition and environmental triggers contribute to disrupted oesophageal epithelial integrity, initiate an innate pro-Th2 immune response and lead to Th2 chronic inflammation with consequent oesophageal dysmotility and fibrosis (196). Airways dysmotility and fibrosis are common features in asthma as well, and therefore EoE may be a representative model for possible similar pathogenic pathways.

Fibrosis is responsible for EoE symptoms such as dysphagia and food impaction. However, children tend to present with less specific symptoms related with oesophageal inflammation and oesophageal dysmotility such as feeding difficulties, gagging and vomiting (196, 197). Confirming what is suspected by examining the differences in symptoms in adult and children, prospective studies suggest that inflammation progresses to fibrosis in the majority of untreated patients (198). Food allergens drive the majority of cases of EoE, but food allergy (FA) in EoE presents differently from IgE mediated FA, in that it appears to start not in infancy but instead seems to be due to a break in tolerance of previously well tolerated common foods such as milk and wheat (199–202). Many lines of evidence suggest that such disruption is due to the epithelial barrier insults and consequent induction of a Pro-Th2 inflammation in genetically predisposed individuals. What such an insult is still largely unknown. Viral infections have often been suspected, but difficult to prove in a disease that needs an endoscopy to be diagnosed. However, a high incidence of EoE (30%) in immunocompetent atopic individuals recovering from Herpes Virus Simplex esophagitis, suggests that viral infection may indeed play a role in EoE (203). If the initial insult has not been clarified, the last 10 years of research have clearly demonstrated the central role of the oesophageal epithelium in driving and maintaining chronic pro inflammatory inflammation in EoE. Eosinophils define the disease in EoE, but the inflammation in EoE is more complex and all the other cell types of atopic inflammation such as basophils, mast cells, IgE producing B cells, innate Lymphocytes type 2 (IL2), invariant natural killer cells (iNKTs) have been shown to be important players (197, 204). This is not surprising considering that like many other diseases, EoE is highly associated with atopic comorbidities and may just be the last step on a slow evolving atopic march (205–208). Food allergens such as milk and wheat and likely environmental allergens are the major drivers of EoE (202, 208–210). Allergens may stimulate Th2 lymphocytes directly, as demonstrated in a small group of patients with milk allergy (209). Although a large proportion of asthmatics have elevated eosinophils and concurrent allergic disease, similar to suffers of EoE, the response to treatments targeting these factors have had difference levels of response in each of these diseases. Differently from other atopic diseases, IgE and eosinophils appear not to be central in the pathogenesis of EoE (208). Indeed, biologics directed against IgE (omalizumab) and eosinophils (anti-IL-5 reslizumab and mepolizumab) have been shown to control asthma in patients with allergic sensitisation and an eosinophilic endotype (211, 212) but have not been proven to be helpful in EoE (213, 214). On the other hand, antibodies with broader Th2 inhibition like antiIL-4/IL-13 or anti-IL-13 seem to be more effective in controlling EoE (215, 216). Due to its genetic predisposition, studies, particularly those involving sibling and twins, have helped to understand how such inflammation develops in EoE (217). Over the years, several genetic loci have been linked to EoE, which confirm the central role of the epithelium in driving EoE and are grouped into three major gene categories: Th2 atopic inflammation, epithelial barrier and fibrosis (218–221).

Several single nucleotide polymorphisms (SNPs) of epithelial genes promoting Th2 inflammation such as eotaxin-3 (*CCL26*) on 7q11.23, thymic stromal lymphopoietin (*TSLP*) on 5q22, and Toll-like receptor 3 (*TLR3*) on 4q35.1, have been connected to EoE by several independent groups (218, 221, 222). Blanchard et al., found a genetic polymorphism in CCL-26 (eotaxin-3), a gene important for eosinophil chemotaxis, present in about 13% of patients with EoE, who overexpressed CCL-26 by 50-foldcompared with healthy controls (218, 221, 223). Similarly, a risk allele (AA) on the TSLP gene has been shown to be correlated with increased epithelial TSLP expression, EoE development and increased mucosal basophils (218, 221, 224). Animal studies have also shown that TSLP is pathogenetic for EoE development as its inhibition prevents EoE development (224). Therefore, TSLP, an epithelial derived cytokine that can strongly induce a Th2 effector response from adaptive and innate cells, appears to be important in EoE pathophysiology like other atopic diseases (225). Other epithelial signalling related polymorphisms such as the *TLR3* SNP (CC or CG at rs3775292), are found more frequently in allergic EoE patients (222).

Th2 inflammation leads to fibrosis in asthmatic patients as well in EoE patients. In EoE, the pro-fibrotic factor transforming growth factor-beta (TGF-β) on 19q13 has been connected with EoE (226, 227). EoE is highly prevalent in patients with connective tissue disorders such as Ehlers- Danlos or Loeys-Dietz or Maran’s Syndromes where dysregulation in TGF-β signalling is well known. Similarly, EoE patients with reduced fibrosis after steroid therapy are more likely to have a certain SNP (the CC genotype at the -509 position) in the TGF-β promoter (226–228). Autosomal dominant Hyper-IgE Syndrome due to dominant-negative STAT3 mutations in which there is an upregulation of TGF-β have higher incidence of EoE (229, 230).

Inflammation is also known to lead to epithelial barrier dysfunction and genes that build the epithelial barrier have been implicated in EoE pathogenesis. These include calpain 14 (*CAPN14*) on chr2p23.1, Filaggrin *(FLG)* on 1q21 and epithelial serine protease inhibitor *SPINK5* on 5q32. Like other atopic diseases such as asthma or eczema, genetic polymorphisms may predispose the epithelium to be more permeable and more vulnerable to damage by the Th2 inflammation. This is thought to increase the amount of contact between antigens and the immune system, and predispose to inappropriate loss of tolerance for these antigens (231). Calpain 14 (*CAPN14*) is a protease in the calpain family that is expressed at the highest level in the esophageal epithelium and upper GI tract. IL-13 induces *CAPN14* expression in the esophageal epithelium with consequent loss of barrier function (218). Netherton’s syndrome, which is caused by a defect in the epithelial serine protease inhibitor SPINK5, has also been described as an EoE risk as well as for severe atopic dermatitis (232, 233).

Although at the moment there is no data that RV or any other virus may induce the epithelium changes that eventually lead to food sensitisation and EoE, it is possible that viruses such as herpes virus or others could act similarly to RV in initiating EoE after infection directly or indirectly through upper airways infection. Indeed, the common cold can involve one or all the sinuses, nasopharynx, oropharynx and larynx. EoE is commonly associated with Eosinophilic Laryngitis and Aerodigestive Dysfunction in children (234). At the molecular level, *CAPN14* is highly expressed in the oesophagus and pharyngeal cells (218). In asthma models, viruses like RV can induce TSLP, and by doing so antagonise tolerance to inhaled antigen (235), or create steroid resistance (176, 236, 237). The cellular constituents are vastly different between the airway epithelium and the oesophageal/gastro-intestinal (GI) epithelium, particularly as the GI epithelium secretes digestive molecules. Nevertheless, they are both endoderm-derived epithelia and both exhibit characteristic allergic features such as inflammation, mucus production, and eosinophil recruitment in response to injury. As a result, it is possible that both the airway epithelium and oesophageal (GI) epithelium may react in the same way to the same stimuli and therefore may also respond to similar therapies. More studies are needed to find any possible connection between viruses and EoE, but these studies and studies on epithelial host response similarities between the two disease processes may be essential and lead to novel common pathogenic mechanism for the induction of multiple inflammatory centered comorbidities.

## Future Perspectives and Therapies in Regards to Epithelial Vulnerability – What Do We Know and Where Do We Go From Here?

It is inherently clear that RV infection is of critical importance in wheezing illnesses leading to asthma development and diagnosis. RV has been shown to have a dysregulatory effect on the barrier function and integrity of the paediatric airway epithelium, particularly in asthmatic children who may already have intrinsic epithelial vulnerabilities. Many of the effects of RV are related to the child’s epithelial host response which is unable to effectively fight the virus (23). Moreover, the plethora of anti-viral and pro-inflammatory responses to RV have been shown to overlap with atopic mechanisms and play a role in other severe allergic diseases including EoE. This highlights the importance of ‘epithelial vulnerability’ with dysregulated epithelial barrier function and integrity at the forefront of allergic disease, and many of them presenting as comorbidities. For example, in one cohort of children with EoE, 59.8% of them also had asthma (205). It also suggests commonality in elucidating effective therapeutics to tackle not only the symptoms of these diseases but their root cause. There are various pharmacological therapies used in the management of wheezing and asthma, EoE and other allergic diseases, although the search continues for more effective therapeutics with improved efficacy, especially considering the health burden for these diseases is large. Particularly, asthma and wheezing exacerbations may still occur regardless of maintenance with ongoing treatment or treatment type, with this echoed in EoE. Most current asthma therapeutics are aimed at treating the visible symptoms to reduce frequency and severity of exacerbations (238, 239). Similarly, as previously mentioned, the focus of treatment for EoE targets resultant fibrosis, but does not prevent or target the underlying cause of the disease. Preventing exacerbations in allergic diseases is challenging as there is a need to first understand the underlying mechanisms. The biological mechanisms as to why allergic diseases such as asthma and EoE occur in children remains to be fully understood and thus, broad therapies including short acting beta agonists, long acting beta agonists, and corticosteroids for asthma; and steroids, biologics directed against IgE and eosinophils and elimination diets for EoE cannot yet be replaced in favour of much more targeted treatment. In addition, some children respond less rapidly to these types of treatment and thus they are less effective (240). Excitingly, studies that have identified novel and re-purposed therapeutics as well as targetable biological pathways that potentially have anti-viral and epithelial barrier integrity aiding effects (61, 241–244). Two in particular include celecoxib, a COX-2 inhibiting non-steroidal anti-inflammatory drug, and azithromycin, a macrolide class antibiotic (61, 243). Celecoxib has been shown to restore wound repair capacity to the airway epithelium and azithromycin has shown to decrease the frequency of asthma exacerbations in adults (61, 243). The effects and full potential of these drugs are still being investigated. Nevertheless, it is postulated that as well as being useful in treating asthma exacerbations they may also be useful in other allergic diseases such as EoE due to their epithelial barrier function-restoring abilities. Furthermore, as TSLP plays an important role in both EoE and RV induced wheezing, Tezepelumab, a TSLP inhibiting agent may help attenuate exacerbations of both diseases though more evidence for this is needed (245). Although these emerging therapeutics are promising, RV infection, particularly RV-C infection in asthma, and epithelial dysregulation and vulnerability in allergic diseases need to be characterised fully in paediatric patients to determine the most effect strategy to improve the quality of life for these children, and thus are of active interest.

This review highlights the growing significance and clinical relevance of an innately vulnerable epithelium in different prevalent allergic-type epithelial diseases, and the effect of viral infection, particularly RV-C infection on airway epithelial barrier function and integrity in paediatric asthma, and viral and injury-induced effects on the oesophageal epithelium in EoE. In asthma specifically, it is postulated that innate epithelial vulnerability further potentiates RV-C infection, due to dysregulated host response to the virus. In addition, RV-C may further potentiate this vulnerability and barrier dysregulation compared to other RV species, leading to a persistent cycle of infection. Furthermore, it is suggested that consecutive or repeated insults to the airway of a child with a vulnerable epithelium culminates in persistent wheezing illnesses and eventually asthma development and diagnosis. This is one current research focus. Subsequently, once this has been identified, therapeutic development pipelines can be developed and potentially be extrapolated to and used in other allergic diseases such as EoE. It is important to compare diseases such as paediatric asthma and EoE as they have parallel disease pathologies and there are lessons that can be learnt from identifying commonalities such as epithelial vulnerabilities in children.

## Author Contributions

RW is the Primary Author of this manuscript. KL, IL, AC, and AK all contributed in equal proportions and have joint Senior Authorship over this manuscript. All authors contributed to the article and approved the submitted version.

## Funding

AK is a Rothwell Family Fellow. Dr Ingrid Laing is funded by NHMRC App#1147630.

## Conflict of Interest

The authors declare that the research was conducted in the absence of any commercial or financial relationships that could be construed as a potential conflict of interest.

## Publisher’s Note

All claims expressed in this article are solely those of the authors and do not necessarily represent those of their affiliated organizations, or those of the publisher, the editors and the reviewers. Any product that may be evaluated in this article, or claim that may be made by its manufacturer, is not guaranteed or endorsed by the publisher.
